# Naproxen-Induced Evans Syndrome

**DOI:** 10.7759/cureus.34910

**Published:** 2023-02-13

**Authors:** Ange Ahoussougbemey Mele, Christopher Chew, Ruben Ruiz Vega, Riaz Mahmood, Riyadh AlRubaye

**Affiliations:** 1 Internal Medicine, Northeast Georgia Medical Center, Gainesville, USA

**Keywords:** hematology, immune thrombocytopenic purpura, autoimmune hemolytic anemia (aiha), naproxen, evans’ syndrome

## Abstract

Evans syndrome is an autoimmune disorder characterized by the simultaneous occurrence of autoimmune hemolytic anemia and immune thrombocytopenic purpura. It can further be classified as primary Evans syndrome when it occurs by itself, or secondary Evans syndrome when it is associated with other autoimmune and lymphoproliferative disorders. Corticosteroids and immunoglobulins are the first-line treatments for primary Evans syndrome, and subsequent options include other immunosuppressive medications. Medical literature provides little information about the triggers of primary Evans syndrome. Knowing such information, however, is essential to recognize, treat and prevent the recurrence of the disease effectively.

We report a 68-year-old female who presented with shortness of breath, cough, bruises, scleral icterus, and dark urine after several days of naproxen therapy for pain. Further workup noted direct antiglobulin test positive for IgG, anemia, and thrombocytopenia. Imaging studies showed deep venous thrombosis. She was diagnosed with Evans syndrome and improved following prompt treatment with corticosteroids, anticoagulants, blood transfusion therapies, and discontinuation of naproxen. The prognosis of Evans syndrome is poor, variable, and characterized by relapses. Early diagnosis and treatment are therefore associated with better prognosis.

This case is critical because it shines a light on one of the major causes of Evans syndrome, reports a practical approach to treating the condition, and paves the way for future research on Evans syndrome. This case is also the first reported naproxen-induced Evans syndrome in the world's literature.

## Introduction

A nationwide retrospective study performed in Denmark reporting 242 patients managed from 1977 to 2017 revealed the annual incidence of Evans syndrome was 1.8/million person-years, and the annual prevalence was 21.3/million persons [1]. Evans et al. described Evans syndrome as a combination of autoimmune thrombocytopenia and AIHA in 1949 and 1951 [2]. The widespread availability and use of non-steroidal anti-inflammatory drugs (NSAIDs) can contribute to the increased incidence of Evans syndrome. However, there needs to be more information regarding the precise mechanism of action of most nonsteroidal anti-inflammatory drugs. Naproxen is a cause of Evans syndrome that has remained unreported and warrants further research. 

## Case presentation

The patient is a 68-year-old female with a past medical history significant for chronic eosinophilia, chronic sinusitis, and environmental allergies who presented initially with shortness of breath and cough. She reported dark-colored urine after taking three doses of doxycycline prescribed for the treatment of sinusitis by her ENT physician. The patient moved to a new house within the last two weeks before arrival. Due to muscle pain, she had been using more cleaning detergents than usual and had taken increased amounts of naproxen over the past two weeks. The patient is also an active user of homeopathic medications but had not taken anything new over the last two years. The patient denied recent travels outside of the United States. A review of systems was positive for a bruise of the left lower extremity bilaterally, shortness of breath, cough, fatigue, and general weakness. Physical findings were notable for scleral icterus and jaundice. 

Upon arrival, the patient was in no acute distress and hemodynamically stable despite a hemoglobin of 6.1 g/dl. Other significant lab values included a platelet count of 62 K/uL, a white blood cell count of 18,000 K/uL, and a total bilirubin of 4.7 mg/dL, with the indirect bilirubin being 4.0 mg/dL. Further hemolytic anemia workup included a haptoglobin of less than one mg/dL, lactate dehydrogenase 1,555 U/L, a large amount of blood in the urine with negative red blood cells, 5.63% reticulocytes, and an immature reticulocyte fraction of 46.3%. Ferritin levels were 221.5 ng/ml, B12 923 pg /ml, folate 23.27 ng/ml, iron 279 ug/dl, and iron saturation of 92%. The direct antiglobulin test was positive for IgG and complement component 3 (C3). Also, the patient had a high eosinophilic percentage on the differential of 20%, and Pappenheimer bodies were present. 

**Figure 1:**
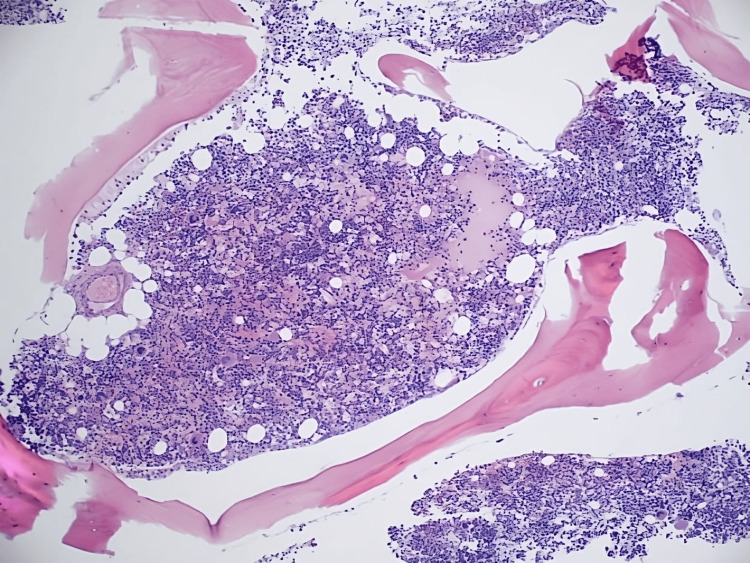
Core biopsy demonstrating hypereosinophilia, and hypercellularity with erythroid and megakaryocytic hyperplasia

**Figure 2:**
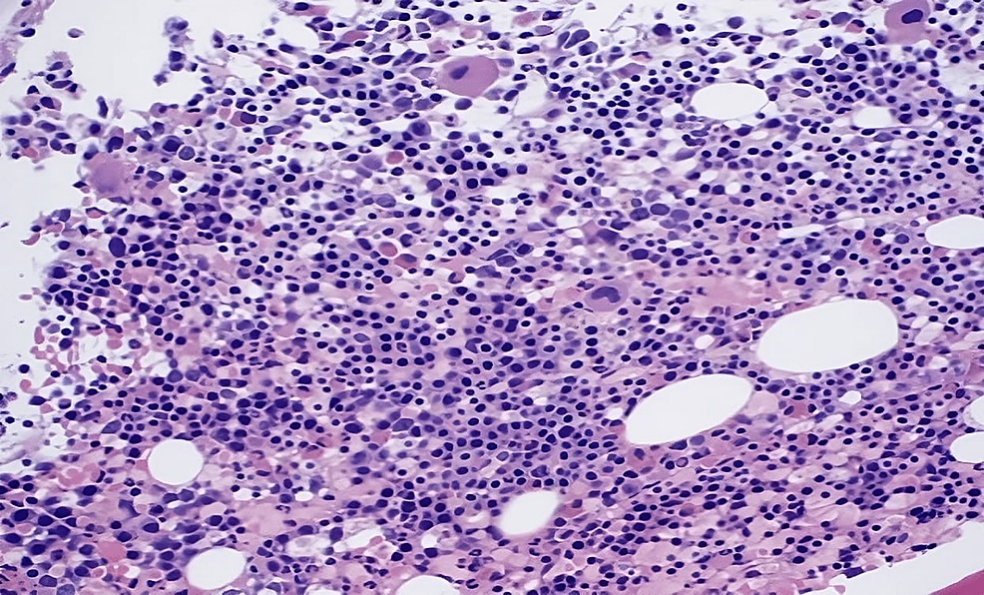
Core biopsy demonstrating hypereosinophilia, and hypercellularity with erythroid and megakaryocytic hyperplasia

Twelve hours after admission, the patient's hemoglobin was 5.4 g/dl, platelets of 34 k/ul with an immature platelet fracture of 14.1%, and a reticulocyte count of 26.73%. We started the patient on corticosteroids resulting in an up-trending hemoglobin and platelet count. A bone marrow biopsy demonstrated hypercellular bone marrow with erythroid hyperplasia, hypereosinophilia, and adequate non-erythroid iron stores (Figures 1, 2). 

**Figure 3:**
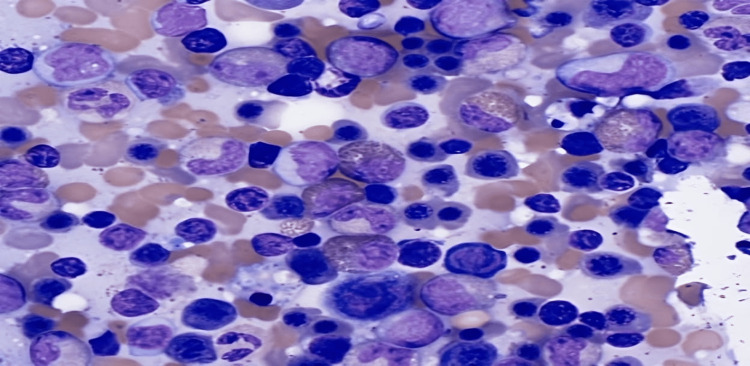
Peripheral smear showing leukocytosis with hypereosinophilia and erythroblastosis, thrombocytopenia, and macrocytic anemia

**Figure 4:**
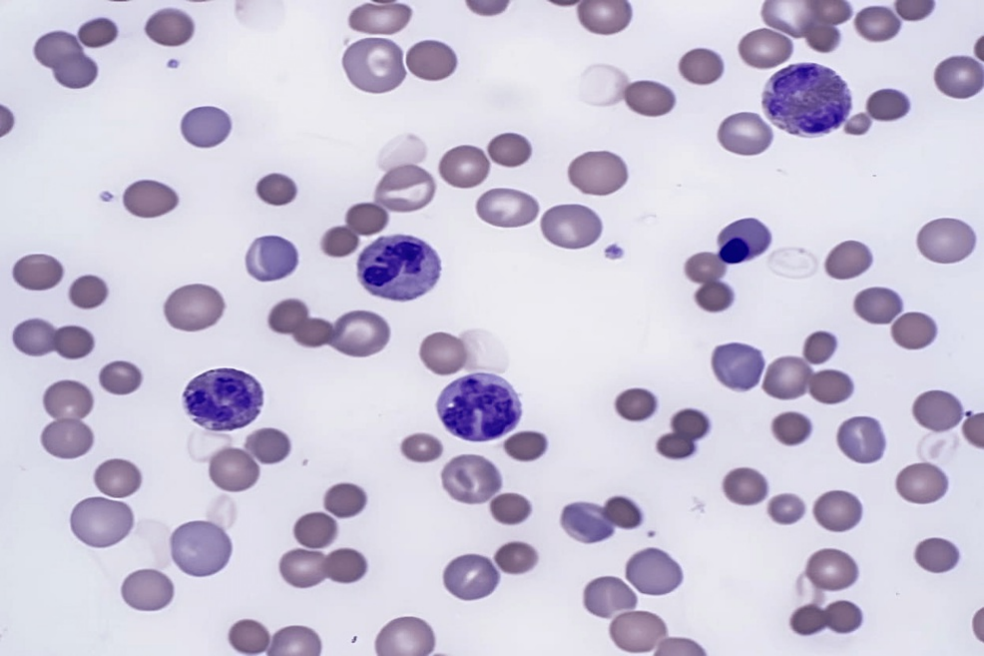
Peripheral smear showing leukocytosis with hypereosinophilia and erythroblastosis, thrombocytopenia, and macrocytic anemia

Peripheral smear was notable for severe anemia and thrombocytopenia with marked macrocytic anemia (Figures 3, 4).

Further workups while in the hospital for AIHA, which was negative, included lead screening, HIV, leukemia and lymphoma panel, hepatitis, fluorescence in situ hybridization (FISH) analysis, flow cytometry, antibodies to extract nuclear antigen, normal complement C3 and C4 levels, lupus anticoagulant, rheumatoid factor, antinuclear antibody (ANA), beta-2 glycoproteins, and Epstein-Barr virus. Positive tests included mycoplasma pneumonia IgG and parvovirus IgG. 

**Figure 5:**
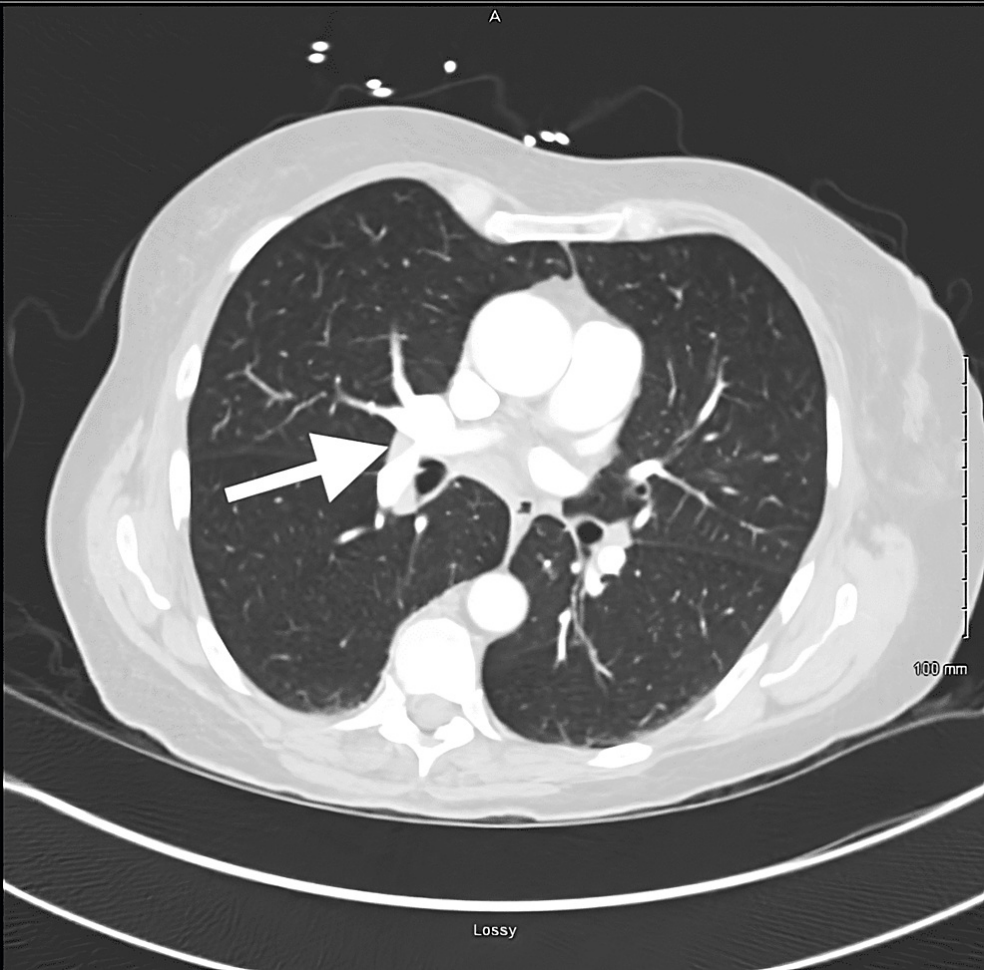
The CTA pulmonary was notable for minimal pulmonary thromboembolism findings with two small adherent filling defects representing thrombi in the right lower lobe pulmonary artery CTA: Computed tomography angiography

During her hospitalization, the computed tomography angiography (CTA) pulmonary was notable for minimal pulmonary thromboembolism findings with two small adherent filling defects representing thrombi in the right lower lobe pulmonary artery (Figure 5). The bilateral venous duplex was notable for deep venous thrombosis of the left peroneal vein. 

The patient's blood counts remained stable, and she left the hospital on prednisone 60 milligrams daily for two weeks with a long taper to be managed by the hematology-oncology outpatient department for the diagnosis of Evan's syndrome, and apixaban 5 milligrams twice daily for three months for deep vein thrombosis and pulmonary embolisms. 

## Discussion

Evans syndrome is a rare autoimmune disorder characterized by autoimmune hemolytic anemia, immune thrombocytopenic purpura (ITP), and a positive direct antibody test for IgG. It can further be classified as a primary idiopathic disorder when it occurs by itself or as a secondary disorder in combination with other autoimmune or lymphoproliferative disorders. It is essential to distinguish between primary and secondary Evans syndrome as the treatment for both differs. As reported by Jaime-Perez et al., recent molecular theories explaining the pathophysiology of Evans syndrome include deficiencies in cytotoxic T-lymphocyte-associated antigen 4, lipopolysaccharide (LPS) response beige-like anchor protein, tripeptidyl peptidase two, and a decrease in the cluster of differentiation (CD)4/CD8 ratio [2]. Moreover, while primary Evans syndrome is a diagnosis of exclusion, the diagnosis of secondary Evans syndrome requires the determination of the baseline disease. A Coomb's positive hemolytic anemia and positive antiplatelet antibodies are essential for Evans syndrome diagnosis. Clinicians rely on patient history, clinical evaluation, and laboratory exams to exclude other causes of AIHA and ITP. Our patient underwent extensive testing to rule out common conditions linked directly with AIHA and ITP. 

The precise cause of Evans syndrome in many patients is usually unknown. Thus, we must stress the importance of clinicians including Evans syndrome as a diagnosis in patients with AIHA and ITP when ruling out other etiologies. Keung et al. reported ace inhibitors as a cause of drug-induced Evans syndrome [3]. Secondary Evans syndrome occurs with common variable immunodeficiency, systemic lupus erythematosus, an autoimmune lymphoproliferative syndrome in non-Hodgkin lymphoma, viral infections such as HIV and hepatitis C, Epstein-Barr virus infection, chronic lymphocytic leukemia and following allogeneic hematopoietic cell transplantation. As per Shaikh et al., the treatment options are different for both primary and secondary Evans syndrome, and pancytopenia is more severe in the setting of Evans syndrome than when presenting with AIHA and ITP alone [4]. 

According to Sanford-Driscoll et al., a review of case reports, clinical studies, and in vitro research has shown that nonsteroidal anti-inflammatory drugs such as mefenamic acid, ibuprofen, sulindac, naproxen, tolmetin, feprazone, and aspirin are well-known culprits of cause autoimmune hemolytic anemia [5]. Some of the most documented cases include mefenamic acid, which causes the condition by an autoimmune mechanism involving an anti-erythrocytic antibody of the IgG class [6], and aspirin which causes Evans syndrome by a complex immune mechanism. Our patient reported an increased naproxen intake in the days preceding her presentation. As such, naproxen is this patient's likely trigger of Evans syndrome. 

Per Barbaryan et al., drug-induced immune hemolytic anemia can be further classified depending on whether antibodies to the drug are present or absent [7]. Drug-dependent antibodies are active only in the presence of the drug, whereas drug-independent antibodies are active in the absence of the drug. If the direct antiglobulin test is positive, an elution test to distinguish drug dependent from drug-independent antibodies is necessary. A negative elution test suggests drug-dependent antibodies since the drug is not present in vitro testing. In the case of drug-independent antibodies, both the direct antiglobulin test and the elution test will be positive. Drug-independent antibodies are almost identical to warm AIHA. The only way to distinguish both is to stop the causative agent and observe for the hematologic response. The treatment of drug-dependent antibodies is the discontinuation of the drug.

In contrast, in the case of drug-independent antibodies, steroids should be added in addition to discontinuing the drug. Our patient's direct antiglobulin test was positive, and eluate evaluation was notable for pan agglutinin and warm autoantibodies. Her hematologic indices improved with the discontinuation of naproxen therapy and treatment with steroids. Naproxen causes Evans syndrome by a drug-independent antibody mechanism of action. 

According to Dhingra et al., bone marrow aspiration is necessary for the workup of Evans syndrome as it allows the clinician to rule out aplastic anemia or infiltrative disorders [8]. The patient's bone marrow core biopsy was notable for hypercellularity with erythroid hyperplasia and mild megakaryocytic hyperplasia. It also demonstrates hypereosinophilia. Prussian blue stain showed adequate non-erythroid iron stores and was negative for increased ring sideroblasts. Furthermore, a peripheral blood smear was notable for leukocytosis with hypereosinophilia, erythroblastosis, thrombocytopenia, and macrocytic anemia. The patient's bone marrow and peripheral blood demonstrated reactive changes in response to AIHA, including erythroid hyperplasia, erythroblastosis, and polychromatophilia (as seen above in Figures 1 and 2). A myeloid neoplasm was not seen based on the bone marrow's morphologic appearance, and flow cytometry showed no evidence of hematopoietic neoplasm. 

According to Norton et al., the first-line treatment for primary Evans syndrome involves corticosteroids, often administered with intravenous immunoglobulins [9]. As per Godeau et al., steroids inhibit the ability of macrophages to clear platelets and erythrocytes, whereas immunoglobulins IgG blocks fragment crystallizable (Fc) gamma receptors on macrophages, thus hindering their effector functions such as phagocytosis [10]. 

When there is no response to first-line therapies, the clinician can use second-line therapy. Rituximab is an anti-CD20 monoclonal antibody used in cases the disease process is refractory to corticosteroid therapy or relapses into Evans syndrome. Furthermore, other efficacious second-line interventions include mycophenolate mofetil which inhibits inosine monophosphate dehydrogenase, thus reducing lymphocyte proliferation. Howard et al. reported its efficacy in treating AIHA and ITP [11]. Cyclosporine is an immunosuppressive medication that acts by inhibiting the activation of T-cells. As reported by Jaime-Perez et al., multiple studies have shown its efficacy in patients who have failed to respond to corticosteroids, intravenous immunoglobulins, and other immunosuppressants. Twenty-six out of the 28 patients treated with cyclosporine had achieved a good response to therapy [2]. Finally, rituximab replaced splenectomy as a second-line treatment due to risks associated with surgery and the heterogeneous response noted in the treatment of Evans syndrome between 0% to 66% [2]. Third-line agents include the alkylating agent cyclophosphamide, the anti-CD52 monoclonal antibody alemtuzumab, and thrombopoietin receptor agonists. As noted above, our patient responded well to first-line therapy with corticosteroids. 

When treating our thrombocytopenic patient, another important question asked was whether or not to use anticoagulants. As postulated by Balitsky et al., the two main premises are that a low platelet count does not protect from thrombosis, and in general, thrombotic complications are more dangerous than bleeding [12]. 

Despite the available treatment options mentioned above, the prognosis of Evans syndrome is poor, variable, and characterized by relapses. A study by Michel et al. described the characteristics and outcomes of Evans syndrome in adults. Sixty-eight adults were selected, amongst which 34 patients had primary Evans syndrome and 34 had secondary Evans syndrome. All patients received corticosteroids, and 50 required a second-line treatment such as splenectomy and rituximab. After a mean follow-up of 4.8 years, 22 patients (32%) were in remission and off treatment. However, 16 patients (24%) died [13].

A cohort study conducted in Denmark noted that the most common causes of death in Evans syndrome were bleeding, infections, and hematological cancer [1]. It accentuates the importance of close follow-up of patients with Evans syndrome upon discharge from the hospital to ensure compliance with the care plan, monitor responses to therapy, and monitor comorbidities. 

## Conclusions

Drug-induced Evans syndrome is a potentially fatal complication of naproxen therapy. It is, therefore, essential to include Evans syndrome in the differential diagnosis of patients presenting with AIHA and ITP. Prompt discontinuation of NSAIDS and treatment with steroids can be lifesaving interventions. Our case reports naproxen as a novel culprit of drug-induced Evans syndrome and proposes an efficacious treatment approach.
